# Functional 4-D clustering for characterizing intratumor heterogeneity in dynamic imaging: evaluation in FDG PET as a prognostic biomarker for breast cancer

**DOI:** 10.1007/s00259-021-05265-8

**Published:** 2021-03-07

**Authors:** Rhea Chitalia, Varsha Viswanath, Austin R. Pantel, Lanell M. Peterson, Aimilia Gastounioti, Eric A. Cohen, Mark Muzi, Joel Karp, David A. Mankoff, Despina Kontos

**Affiliations:** 1 Department of Bioengineering, University of Pennsylvania, Philadelphia, PA USA; 2 Department of Radiology, University of Pennsylvania, Rm. D702 Richards Bldg. 3700 Hamilton Walk, Philadelphia, PA 19104 USA; 3 Department of Radiology, University of Washington, Seattle, WA USA

**Keywords:** Intratumor heterogeneity, Breast cancer, Dynamic PET, Imaging markers

## Abstract

**Purpose:**

Probe-based dynamic (4-D) imaging modalities capture breast intratumor heterogeneity both spatially and kinetically. Characterizing heterogeneity through tumor sub-populations with distinct functional behavior may elucidate tumor biology to improve targeted therapy specificity and enable precision clinical decision making.

**Methods:**

We propose an unsupervised clustering algorithm for 4-D imaging that integrates Markov-Random Field (MRF) image segmentation with time-series analysis to characterize kinetic intratumor heterogeneity. We applied this to dynamic FDG PET scans by identifying distinct time-activity curve (TAC) profiles with spatial proximity constraints. We first evaluated algorithm performance using simulated dynamic data. We then applied our algorithm to a dataset of 50 women with locally advanced breast cancer imaged by dynamic FDG PET prior to treatment and followed to monitor for disease recurrence. A functional tumor heterogeneity (FTH) signature was then extracted from functionally distinct sub-regions within each tumor. Cross-validated time-to-event analysis was performed to assess the prognostic value of FTH signatures compared to established histopathological and kinetic prognostic markers.

**Results:**

Adding FTH signatures to a baseline model of known predictors of disease recurrence and established FDG PET uptake and kinetic markers improved the concordance statistic (C-statistic) from 0.59 to 0.74 (*p* = 0.005). Unsupervised hierarchical clustering of the FTH signatures identified two significant (*p* < 0.001) phenotypes of tumor heterogeneity corresponding to high and low FTH. Distributions of FDG flux, or Ki, were significantly different (*p* = 0.04) across the two phenotypes.

**Conclusions:**

Our findings suggest that imaging markers of FTH add independent value beyond standard PET imaging metrics in predicting recurrence-free survival in breast cancer and thus merit further study.

**Supplementary Information:**

The online version contains supplementary material available at 10.1007/s00259-021-05265-8.

## Introduction

Cancer heterogeneity is well-established, with inter- and intratumor manifestations recognized as key prognostic and predictive factors [1–5]. Increased intratumor heterogeneity is associated with adverse outcomes [6]. Tumor progression driven by aggressive cell subpopulations has been shown to be a mechanism for recurrence and therapy resistance [5]. Quantitative characterization of intratumor heterogeneity could allow for novel precision prognostic and predictive indicators.

Molecular and functional imaging modalities permit 4-D sampling of disease burden, capturing both spatial and temporal information that could illuminate various physiologic behaviors. Dynamic positron emission tomography (PET) imaging can quantify specific facets of tumor molecular biology [4, 7, 8] and can provide information beyond that of static imaging [9, 10]. Dynamic PET imaging of the glucose analog, ^18^F-fluorodeoxyglucose (FDG), can provide simultaneous information on substrate delivery and metabolism [9]. Current clinical characterization of malignant lesions using PET imaging largely utilizes qualitative descriptors [5] and quantitative measures based on static radiotracer uptake (e.g., SUVmax) [11].

The emerging field of radiomics has introduced multi-parametric imaging features extracted with high-throughput computational analysis [12–15]. Previous work by Eary et al. quantified spatial heterogeneity of radiotracer uptake in static PET imaging and demonstrated improved prognostic performance over established clinical markers [16]. Stoyanova et al. identified sub-regions within pre-clinical dynamic contrast enhanced (DCE)-MRI images of prostate tumors [17]. Similarly, Cherezov et al. identified tumor habitats using established radiomic texture features [18]. While such studies demonstrate the prognostic potential of characterizing 3-D spatial heterogeneity, and are in line with studies showing differential physiologic functionality across the whole tumor [19, 20], these studies do not fully utilize the combined spatial and kinetic (e.g., 4-D) heterogeneity information available using imaging probe kinetics from modalities with high temporal imaging resolution.

The advantages of utilizing kinetic information from dynamic PET imaging have been demonstrated in breast cancer. Previous studies have demonstrated predictive improvement when FDG delivery (K_1_) and FDG flux (Ki), in combination with [^15^O]-water imaging, were utilized with static SUV measures as markers for neoadjuvant chemotherapy response in patients with locally advanced breast cancer [9, 21, 22]. However, these conventional kinetic parameters derived from FDG PET imaging at baseline alone were unable to show association to disease-free survival, likely limited by being derived from the most metabolically active portion of the tumor, and therefore not fully capturing intratumor heterogeneity [9].

We have developed a method to characterize 4-D functional tumor heterogeneity (FTH) by capturing aspects of both spatial and kinetic tumor heterogeneity seen in dynamic imaging. The improved dynamic sampling and molecular specificity available in dynamic PET as compared to other imaging modalities may allow for non-invasive, novel prognostic, and predictive markers to characterize tumor molecular biology. The developed method is agnostic to the specific radiotracer utilized and does not depend on complex kinetic modeling assumptions. Instead, the approach is data driven in terms of identifying intrinsic 4-D patterns of molecular tumor heterogeneity. We present proof-of-principle by applying our algorithm on dynamic FDG PET imaging scans of primary locally advanced breast cancer. We also investigate the role of imaging signatures as a prognostic biomarker for locally advanced breast cancer and the improved predictive value of FTH characterization compared to standard dynamic and static analytic methods for FDG PET.

## Materials and methods

### Radiomic functional intratumor (Rad-FIT) clustering: development and validation

#### Dynamic PET simulated image phantoms

**Fig. 1:**
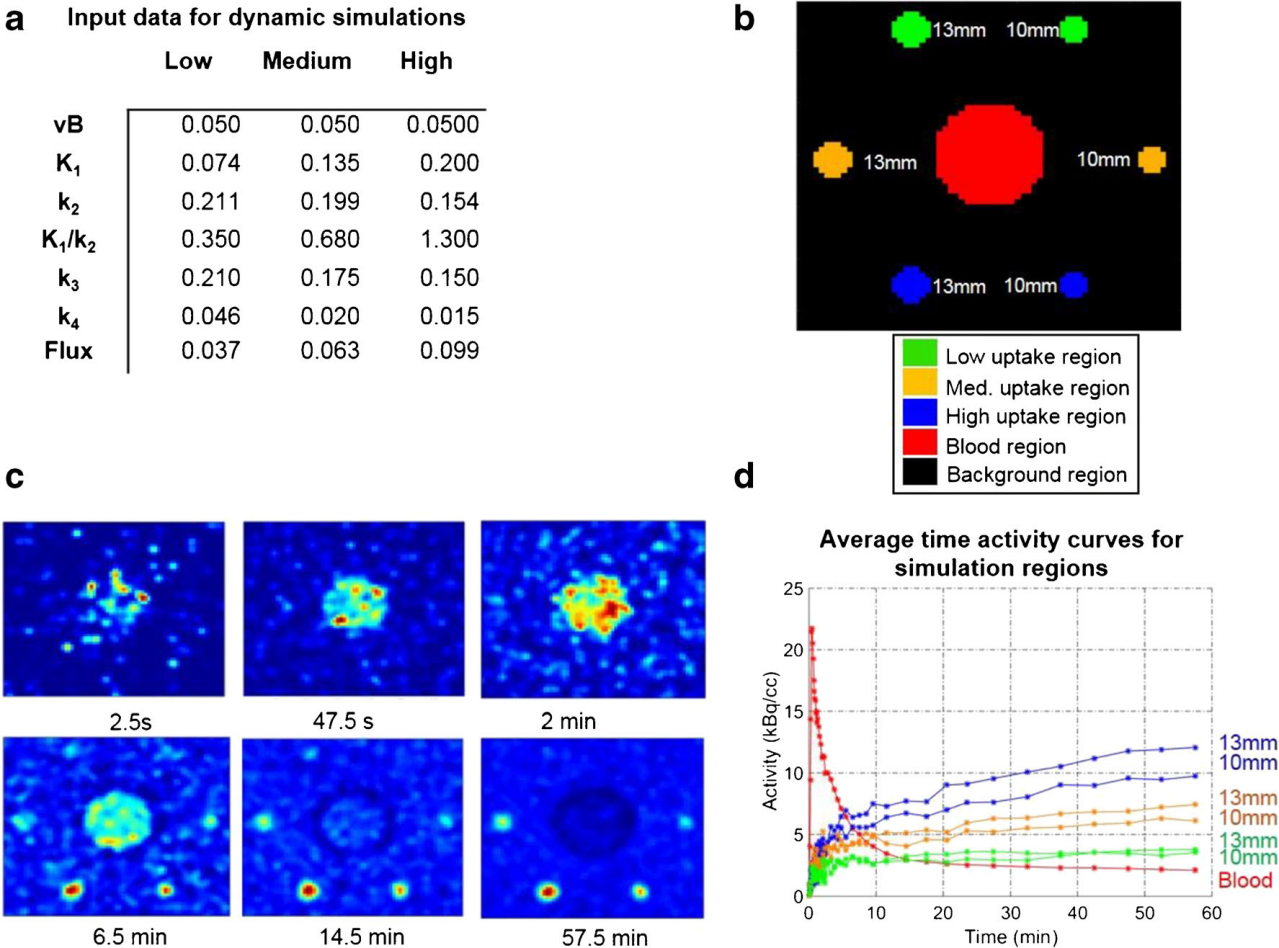
Dynamic FLT-PET simulation images used for method development and validation. **a** Input data used to generate dynamic simulations. **b** Labels for all regions in simulated image. **c** Center slice images from representative simulation frames. **d** Average TACs for simulation sphere and blood curve regions

Algorithm development and training were carried out using simulated 4-D PET data. With the goal of developing a method with broad applicability, we chose to perform these algorithm trainings using a different tracer and tumor type than FDG and breast cancer, but with broadly similar tracer kinetics. Simulated dynamic PET images based on data from fluorothymidine (^18^F-FLT) (FLT) PET [23] were utilized for method development and validation. FLT was chosen as a representative tracer with kinetics similar to other trapped cancer-relevant radiotracers to assess the generalizability of our method. All simulations were done using Geant4 Application for Tomographic Emission (GATE) [24]. The scanner simulations were based on the PennPET Explorer [25] with 70-cm axial field of view. Based on prior human studies of FLT data from patients with lung cancer [23], kinetic parameters were selected to emulate low, medium, and high K_FLT_ lesions (ml/cm^3^/s), and the blood input curve was derived from an FLT PET patient dataset and fit to a tri-exponential model. Details on simulated image generation have been previously described [26]. The simulated images were cropped to a region-of-interest (ROI) comprising the simulated regions and surround background area to a total size of 64 × 69 × 9 voxels × 45 frames. The simulated images consisted of two regions modeling low tracer uptake (10-mm and 13-mm sphere diameter), two regions modeling medium tracer uptake (10-mm and 13-mm sphere diameter), two regions modeling high tracer uptake (10-mm and 13-mm sphere diameter), a blood curve region, and a background region (Fig. 1).

#### Radiomic functional intratumor (Rad-FIT) clustering algorithm

To accurately assess both the spatial and temporal behavior seen in breast tumors, radiomic functional intratumor (Rad-FIT) clustering was developed as a 4-D-segmentation method to account for voxel similarity in the time domain as well as weight spatial contiguity. The temporal signal of an ROI over dynamic PET scans is first summarized using functional principal component analysis (FPCA), with each voxel represented using functional principal components (FPC) capturing greater than 85% of the variance seen in its dynamic behavior. Use of FPCA allows for the dimensionality reduction of time series data for each voxel while retaining the variance of its temporal information.

A Markov Random Field [27] (MRF) segmentation paradigm is then applied to the voxel data for a learning-based method which incorporates spatial interactions between voxels allowing for a 3 × 3 × 3 voxel neighborhood surrounding each voxel to influence segmentation. Initialization of mean and standard deviation values for each label *k* is performed using K-means clustering with a predetermined number of *k* labels. The final segmentations are then determined using the expectation maximization algorithm [28]. Details regarding this methodology can be found in the Supplementary materials (Supplementary information, Supplemental Fig. 1, Supplemental Table 1).

#### Rad- FIT evaluation and comparison to existing unsupervised clustering methods

To evaluate the performance of segmenting spatially contiguous regions of heterogeneous tracer activity with Rad-FIT, the following segmentation assessments were performed.

First, the improved value of summarizing temporal information from dynamic voxel behavior using FPCs was evaluated. The segmentation performance of Rad-FIT when simulation voxels were represented by their original time activity curves was compared to the performance when simulation voxels were represented by their FPC scores.

The segmentation performance of Rad-FIT was then evaluated for its ability to segment the high, medium, and low uptake 13-mm spheres from each sphere’s surrounding background region, respectively. All segmentation performances were compared against the performance of established unsupervised segmentation algorithms including K-means clustering, hierarchical clustering, and spectral clustering [29]. Lastly, Rad-FIT segmentation performance was compared to the highest performing unsupervised clustering algorithm as outlined above when segmenting the simulated image into five distinct regions: background, blood, and low, medium, and high sphere regions. All segmentation performances were evaluated using the Dice score [30] and Jaccard index, averaged over ten replicates. Both the Dice score and Jaccard index are established statistical metrics used to determine the degree of overlap between the true regions and resulting regions from the segmentation algorithm. Both Dice scores and Jaccard indices include values ranging from 0 to 1, with a value of 1 indicating perfect similarity between true and segmented regions.

### Functional tumor heterogeneity signature as a prognostic biomarker for dynamic FDG PET imaging of breast cancer

#### Study cohort

To investigate the role of intratumor segmentation when characterizing functional heterogeneity; the prognostic value of functional tumor heterogeneity imaging signatures was explored on a previously published data set where serial dynamic FDG PET was shown to be predictive of response and recurrence using standard static uptake and kinetic analysis [9, 21, 22]. The goal was to test functional tumor heterogeneity imaging signatures extracted from dynamic FDG PET scans of women with locally advanced breast cancer imaging prior to treatment and compare their predictive value to standard approaches.

We used an anonymized data set consisting of women presenting at the University of Washington Breast Cancer Specialty Center with histologically confirmed breast carcinoma who underwent dynamic FDG PET imaging prior to neoadjuvant chemotherapy and were followed for disease recurrence. The research protocol was approved by the institutional review board and patients studied provided informed consent prior to imaging and follow-up. The data set for this analysis was taken from a study first reported for 35 patients [21]. An additional 30 patients were later studied and added to a follow-up report of the data [9, 22]. From this pooled data set of 65 women with complete baseline dynamic FDG PET scans who also completed neo-adjuvant chemotherapy and post-therapy surgery, two women were excluded for electing not to receive chemotherapy, three women were excluded for electing for medical care elsewhere, four patients were excluded for being unwilling to undergo mid-therapy imaging, two patients were excluded due to distant disease, and one patient was excluded due to little or no tracer uptake upon pre-therapy examination resulting in a total of 53 women. Of these, two women were excluded due to image artifacts and one woman was excluded due to incomplete survival information, resulting in a total of 50 women included in this study. Dynamic FDG PET images from these 50 women comprised our study sample reported here. Details of the patient population have been previously described [22].

Each woman had undergone 60-min dynamic FDG PET centered over the breast prior to neoadjuvant chemotherapy and breast surgery. All women were imaged in the supine position and no positioning devices for immobilization were utilized. Women were infused with 218–396 MBq of FDG over 2 min in a 7–10 mL volume, with an intended injected dose of 370 MBq. Images for all women were acquired on an Advanced Tomograph (General Electric Medical Systems, Waukesha, WI) using the same image acquisition protocol. Dynamic images were acquired (25 image frames: 1-min pre-injection frame, 4 × 20 s, 4 × 40 s, 4 × 40 s, 4 × 1 min, 4 × 3 min, 8 × 5 min). Images were reconstructed into 35 × 128 × 128 voxel matrices with a spatial resolution of 10–12 mm [21]. Clinical information collected as part of the study included hormone receptor (HR) status consisting of estrogen receptor (ER) and progesterone receptor (PR), human epidermal growth factor receptor 2 (HER2), clinical stage, tumor size, proliferation (Ki67), pathologic complete response (pCR), axillary lymph node (ALN) positivity, and age at diagnosis (Supplemental Table 2). In the study, recurrence free survival (RFS) was tracked for each patient, defined as date of known recurrence, date of death, date of most recent clinical follow-up with no evidence of disease, following the patient’s date of surgery. Patients received standard of care follow-up including routine period imaging of CT scans, blood marker analysis (CA2729), and follow-up visits to check for symptoms.

Established ROI-based measures of uptake and kinetics for dynamic FDG PET—summed imaged standardized uptake value (SUV), and the kinetic parameters of FDG blood-to-tissue transport (K_1_) and FDG trapping flux (Ki)—were calculated based on kinetic modeling of dynamic data for each woman and have been previously reported [9, 21]. These kinetic parameters were measured for each tumor from a 1.5-cm-diameter circle VOI surrounding the area of maximal tumor FDG uptake seen on the 30–60-m summed image.

#### Functional tumor heterogeneity (FTH) signature extraction

A 3-D bounding region surrounding each unifocal lesion was manually identified by a radiologist blinded to the outcome of each patient using the final of the summed FDG images for the 25 imaging frames (30–60 min post-injection) and guided by ROIs previously used for extraction of SUV, K_1_, and Ki for consistency. An established segmentation approach was applied to the TACs generated from the 25 imaging frames of each voxel within the bounding region to segment the tumor from its surrounding background [31].

Within the segmented 3-D tumor region, Rad-FIT clustering was applied to segment each tumor region into three, spatially constrained sub-regions with distinct functional behavior. Three sub-regions were selected based on the rationale that there are currently three major subtypes of breast tumors broadly recognized: hormone receptor positive, HER2 positive, and triple negative [2]. The three sub-regions within each tumor were ranked in the order of descending mean value of the first FPC to allow for consistent comparisons across tumors.

The resulting Rad-FIT clustering within each tumor was summarized using metrics describing sub-region compactness and separation. These metrics were chosen to summarize how well the functional behavior of each tumor will cluster into three groups and to allow for comparisons of intratumor heterogeneity across women. Compactness was measured using the between cluster sum of squares (BCSS) scaled by the total sum of squares (TSS).:

1 $$ \frac{\sum_{k=1}^K{\left(\overline{x_k}-\overline{X}\right)}^2}{\sum_{i=1}^N{\left({x}_i-\overline{X}\right)}^2} $$

where *K* represents the 3 sub-regions and *N* is the total number of voxels within each tumor. The separation between sub-regions was determined using the Bhattacharya distance [32] to calculate the distance between FPC distributions of two sub-regions (*ϕ*). Use of this distance allows for a similarity measure between the distributions of FPC values within two sub-regions.

**Fig. 2:**
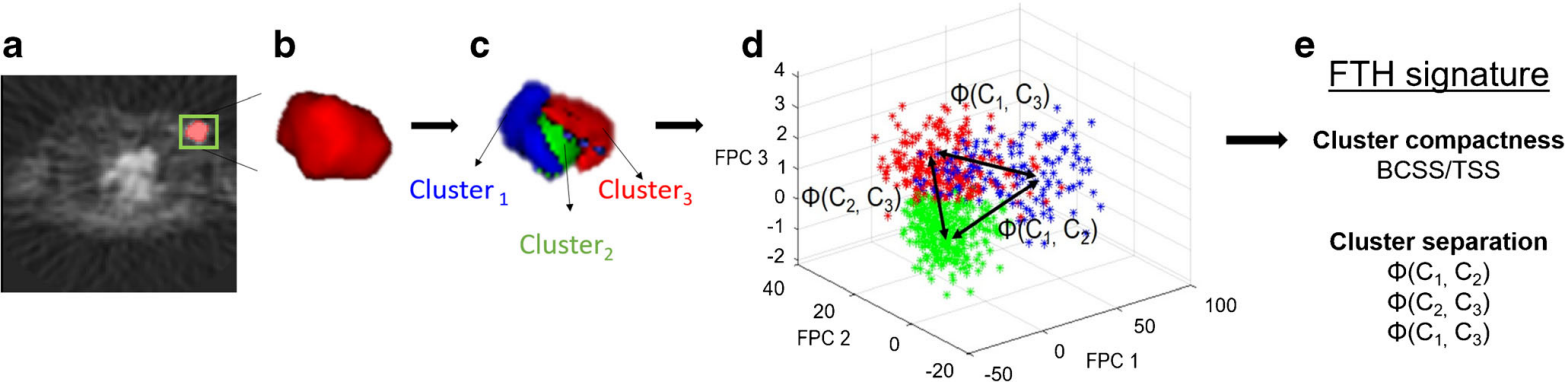
FTH signature extraction. **a** 3-D tumor region identified by a radiologist shown in green. **b** Automated 3-D segmentation of tumor from background with pixels represented using time activity curves. **c** Rad-FIT clustering performed, identifying three, spatially contiguous sub-regions. **d** Cluster compactness and cluster separation distances are calculated to form features in FTH signature. **e** Intratumor heterogeneity summarized using the FTH signature

Based on the definitions above, a total of four features summarizing intratumor heterogeneity from Rad-FIT clustering results were extracted to form an FTH signature (Fig. 2): (1) BCSS/TSS, (2) distance between sub-region 1 and 2 (*ϕ*(1,2)), (3) distance between sub-region 2 and 3 (*ϕ*(2,3)), and (4) distance between sub-region 1 and 3 (*ϕ*(1,3)).

BCSS/TSS provides a measure of how compact the resulting clusters are; the more compact each cluster is, the greater heterogeneity between the identified sub-regions. Calculating the distance between the distributions of FPC values of two sub-regions provides a metric for how separated the clusters are; a greater distance between sub-regions indicates greater heterogeneity within the whole tumor region. This FTH signature can be used to interpret how distinct the three identified sub-regions are within each tumor. As such, the average value of the FTH signature, or FTH signature index, can provide a metric for intratumor heterogeneity across tumors.

#### Statistical analysis: evaluation of FTH signatures as a prognostic biomarker

Our goals in this proof of principal study were to test the prognostic value of FTH signatures from dynamic breast cancer FDG PET and to assess for incremental value compared to standard clinical parameters and conventional FDG PET static and dynamic analysis measures used in prior published analyses. FTH signatures were first *z* score normalized across all women. Time-to-event analysis was then used to assess the prognostic value of the FTH signatures in predicting recurrence-free survival (RFS). To this end, a threefold cross validated (CV) Cox proportional hazards model was used to compare improved prognostic discriminatory capacity over baseline models of established prognostic factors consisting of ER status, PR status, tumor size, pCR, and ALN positivity and kinetic parameters consisting of the SUV, K_1_, and Ki. These prognostic factors were chosen based on the available data as well as the intent to compare analysis results to prior published data [9, 21, 22].

Model performance was evaluated using an averaged C-statistic over the test sets for all three folds and the log-likelihood statistical test.

The prognostic value of the FTH signature was evaluated via Kaplan-Meier survival analysis using each patient’s risk core, dichotomizing patients into high- and low-risk groups. The risk score for each patient was defined as the patient’s FTH signature weighted by the corresponding coefficients from each of the three test sets from a threefold cross validated model for each covariate in the FTH signature [12, 33]. Risk scores generated from baseline features of ER status, PR status, tumor size, pCR, and ALN positivity and from baseline and kinetic features were also assessed. Statistical significance of Kaplan-Meier stratification was evaluated using the Log Rank Test.

Lastly, an exploratory unsupervised hierarchical clustering was performed on the extracted FTH signatures from each woman. The resulting *c* clusters obtained from the hierarchical clustering algorithm were interpreted as *c* intrinsic FTH phenotypes seen in this study population. The optimal number of stable FTH phenotypes was determined using consensus clustering [34]. Statistical significance of the identified, stable FTH phenotypes was evaluated using the SigClust method [35]. The distribution of histopathologic and kinetic prognostic covariate values across women assigned to each of the FTH phenotypes was assessed using chi-square tests for categorical biomarkers and one-way analysis of variance test for continuous biomarkers.

## Results

### Rad-FIT evaluation and comparison to existing unsupervised clustering methods

Average signal to background ratios for the low, medium, and high uptake spheres were 1.78, 6.95, and 11.89, respectively. The segmentation performance of Rad-FIT clustering was compared against existing unsupervised segmentation methods, *K*-means clustering, hierarchical clustering, and spectral clustering, when applied to the simulated images with varying regions of known uptake value and location.

When segmenting low, medium, and high uptake spheres from its surrounding background region, segmentation performance for all segmentation methods improved when simulation voxels were represented by the FPCs capturing greater than 85% of TAC variability as opposed to using the TACs. Additionally, Rad-FIT clustering demonstrated the highest segmentation performance when segmenting low, medium, and high uptake sphere regions from its surrounding backgrounds when evaluated using the Dice score (Table 1) and Jaccard index (Supplemental Table 3).

**Table 1:** Average segmentation performance over ten replicates evaluated using the Dice scores when segmenting low, medium, and high uptake simulated sphere regions from surrounding backgrounds. Standard deviation in parentheses

		Dice scores			
Segmentation region	Voxel representation	Hierarchical clustering	Spectral clustering	*K*-means clustering	Rad-FIT clustering
Low uptake sphere	Time activity curves	0.15 (0)	0.04 (0.001)	0.13 (0.06)	0.24 (0.07)
	FPC	0.67 (0)	0.16 (0.03)	0.65 (0.02)	0.70 (0.01)
Medium uptake sphere	Time activity curves	0.77 (< 0.001)	0.06 (0.01)	0.44 (0.4)	0.73 (< .001)
	FPC	0.78 (0)	0.20 (0.18)	0.84 (0.03)	0.85 (0)
High uptake sphere	Time activity curves	0.72 (0)	0.18 (0.07)	0.52 (0.40)	0.66 (0)
	FPC	0.83 (0)	0.17 (0.03)	0.84 (0)	0.86 (0)

**Fig. 3:**
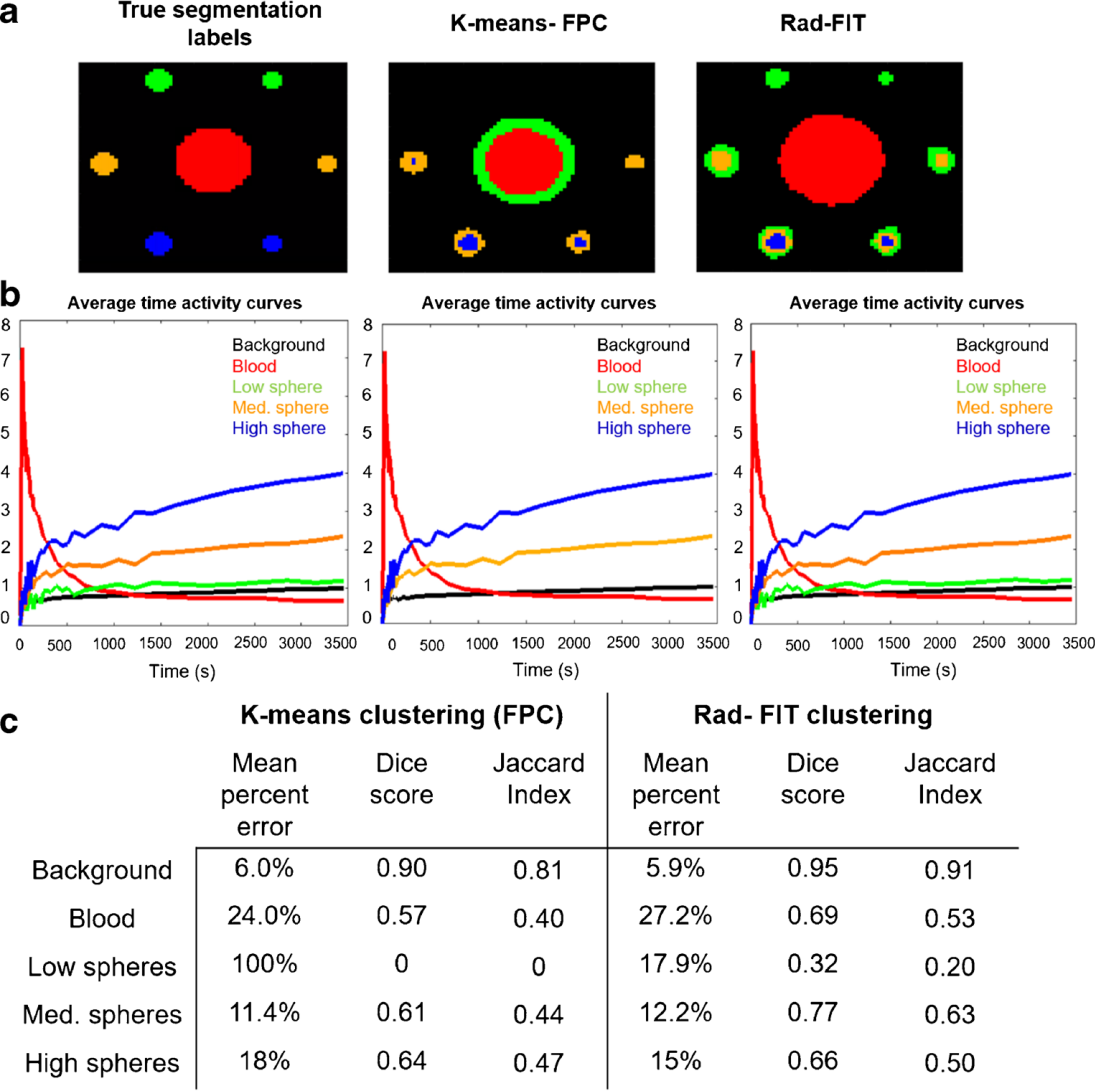
**a** Representative center slice image of true segmentation labels (left), segmentation results using *K*-means clustering (middle), and segmentation results using Rad-FIT clustering (right) when segmenting simulation images into five classes: background, blood, and low, medium, and high uptake spheres. **b** Average time activity curves for true simulation regions (left), *K*-means clustering identified regions (middle), and Rad-FIT clustering (right) identified regions. **c** Mean percent error, generalized dice score, and Jaccard indices for segmentation results. The *K*-means clustering algorithm fails to segment the low uptake spheres from the background region, and instead segments the blood region to two separate classes

The *K*-means clustering algorithm had the second highest segmentation performance across the evaluated unsupervised clustering algorithms. This algorithm was subsequently used to compare performance against the Rad-FIT clustering algorithm to segment the simulated image into five regions: background, blood, low uptake spheres, medium uptake spheres, and high uptake spheres. The Rad-FIT clustering algorithm was able to segment the low uptake spheres from its surrounding background region and identify the five regions of distinct tracer uptake (Fig. 3). In comparison, the *K*-means clustering algorithm was unable to identify and segment the low uptake spheres as a distinct region from its surrounding background. Overall, the Rad-FIT clustering algorithm demonstrated improved segmentation over the K-means clustering algorithm as demonstrated by decreased mean percent error and increased Dice score and Jaccard index for each individual region’s segmentation as well as segmenting the overall simulated image. Due to this improved segmentation performance, the Rad-FIT clustering algorithm was utilized as the unsupervised clustering method towards characterizing functional tumor heterogeneity as a prognostic biomarker for women diagnosed with locally advanced breast cancer.

### Evaluation of the FTH signature as a prognostic biomarker

Of the 50 women included in the data set selected from prior studies for evaluation of FTH signatures as a prognostic biomarker, 17 women (34%) had recurrence events. A total of 47 (94%) women were diagnosed with infiltrating ductal carcinoma, and 3 (6%) women were diagnosed with infiltrating lobular carcinoma.

Of the non-recurrent cases, 58% were ER positive, 52% were PR positive, and 18% were HER2 positive. Of the recurrent cases, 59% were ER positive, 59% were PR positive, and 35% were HER2 positive (Supplemental Table 2).

**Fig. 4:**
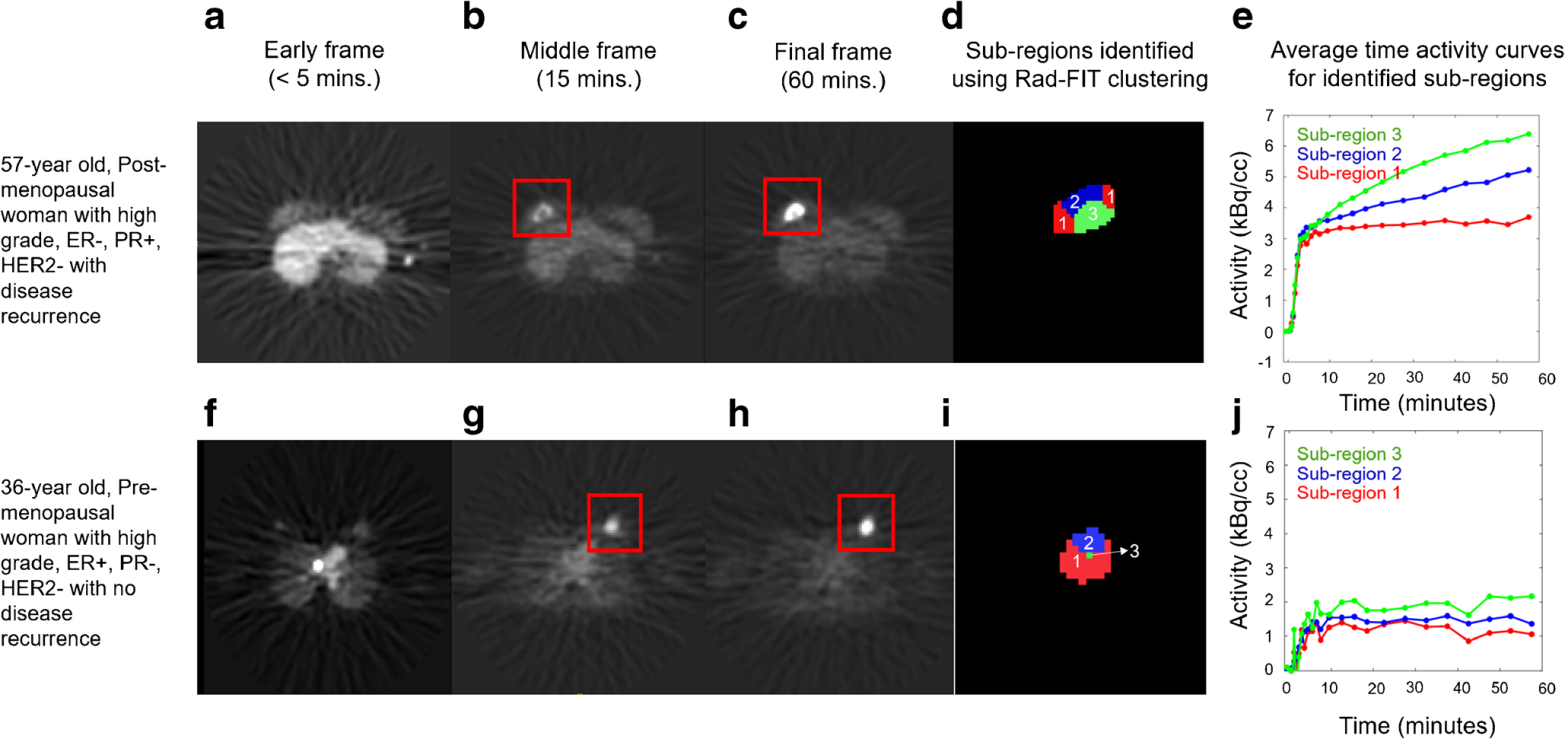
Representative images for the primary tumor of two women diagnosed with locally advanced breast cancer with future tumor recurrence (top) or tumor non-recurrence (bottom). **a** Representative slice from an early frame at less than 5 min after tracer injection, (**b**) representative slice from middle frame at 15 min after tracer injection, and **c** representative slice from final frame taken at 60 min after tracer injection of a 57-year old, post-menopausal woman with a high grade-, ER−, PR+, HER2− tumor who had disease recurrence upon follow-up (top). **d** Three sub-regions identified using Rad-FIT clustering labeled as region 1 (red), 2 (blue), and 3 (green), and **e** average TACs for each identified sub-region. **f** A representative slice from an early frame at less than 5 min after tracer injection, **g** slice from middle frame at 15 min after tracer injection, and **h** representative slice from final frame taken at 60 min after tracer injection of a 36-year old, pre-menopausal woman with a high-grade, ER+, PR−, HER2− tumor with no disease recurrence (bottom). **i** Three sub-regions with distinct 4-D behavior identified using Rad-FIT clustering labeled as region 1 (red), 2 (blue), and 3 (green), and **j** average time activity curves for each identified sub-region

Representative tumor images after Rad-FIT clustering demonstrate intratumor heterogeneity within breast tumors (Fig. 4). Tumors with increased intratumor heterogeneity can be identified as having sub-regions with distinct time activity curve behaviors, while tumors with decreased intratumor heterogeneity display little distinction between the time activity curve behavior of the identified sub-regions.

**Fig. 5:**
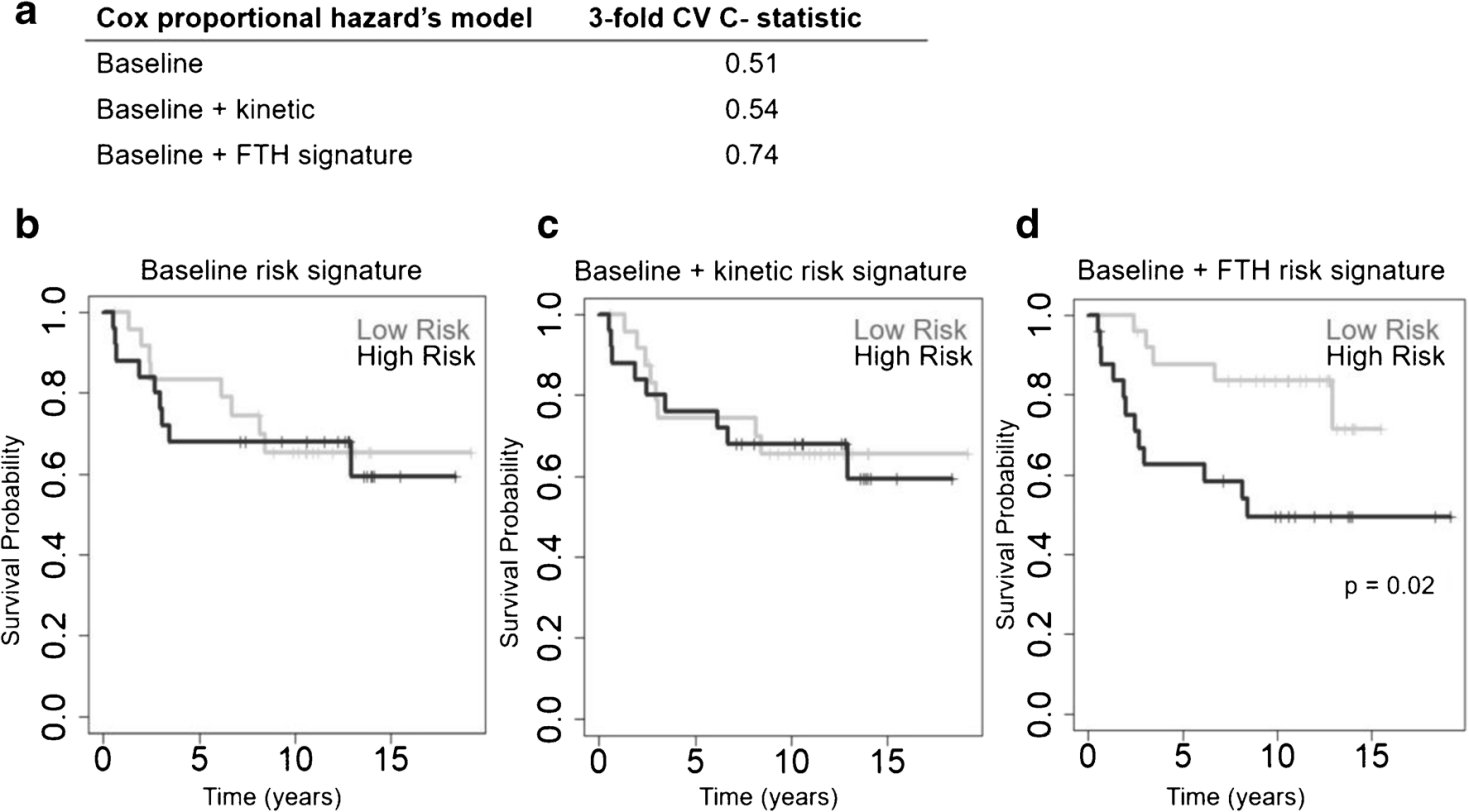
**a** Cross-validated *c* scores and Kaplan-Meier survival curves for baseline (ER status, PR status, tumor size at baseline, pCR, and ALN positivity), baseline plus kinetic (ER status, PR status, tumor size at baseline, pCR, ALN positivity, SUV, K_1_, Ki), and baseline plus FTH signature models. Kaplan-Meier curves generated when patients are stratified by risk scores generated from **b** the baseline model, **c** baseline plus kinetic features model, and **d** baseline plus FTH signature model

As expected, in a full multivariate Cox proportional hazards model after adjusting for ER status, PR status, tumor size, pCR, and ALN positivity (Supplemental Table 4), *ϕ*(1,2) and *ϕ*(2,3) were associated with disease-free survival (Supplemental Table 5). A baseline, threefold CV Cox proportional hazards model consisting of ER status, PR status, tumor size, pCR, and ALN positivity resulted in a mean C-statistic of 0.51 when predicting RFS. Adding SUV, K_1_, and Ki parameters to the baseline model resulted in a mean CV C-statistic of 0.54. Adding the FTH signature to the baseline model improved the mean CV C-statistic to 0.74 (*p* < 0.01) (Fig. 5a).

Dichotomizing patients into low- and high-risk groups based on the baseline model risk scores (Fig. 5b) and baseline plus the kinetic model risk scores (Fig. 5c) demonstrated no statistically significant separation between Kaplan-Meier curves. Patient dichotomization into low- and high-risk groups based on the baseline plus FTH signature risk scores (Fig. 5d) resulted in a statistically significant separation between Kaplan- Meier curves (*p* < 0.05) for RFS probability.

**Fig. 6:**
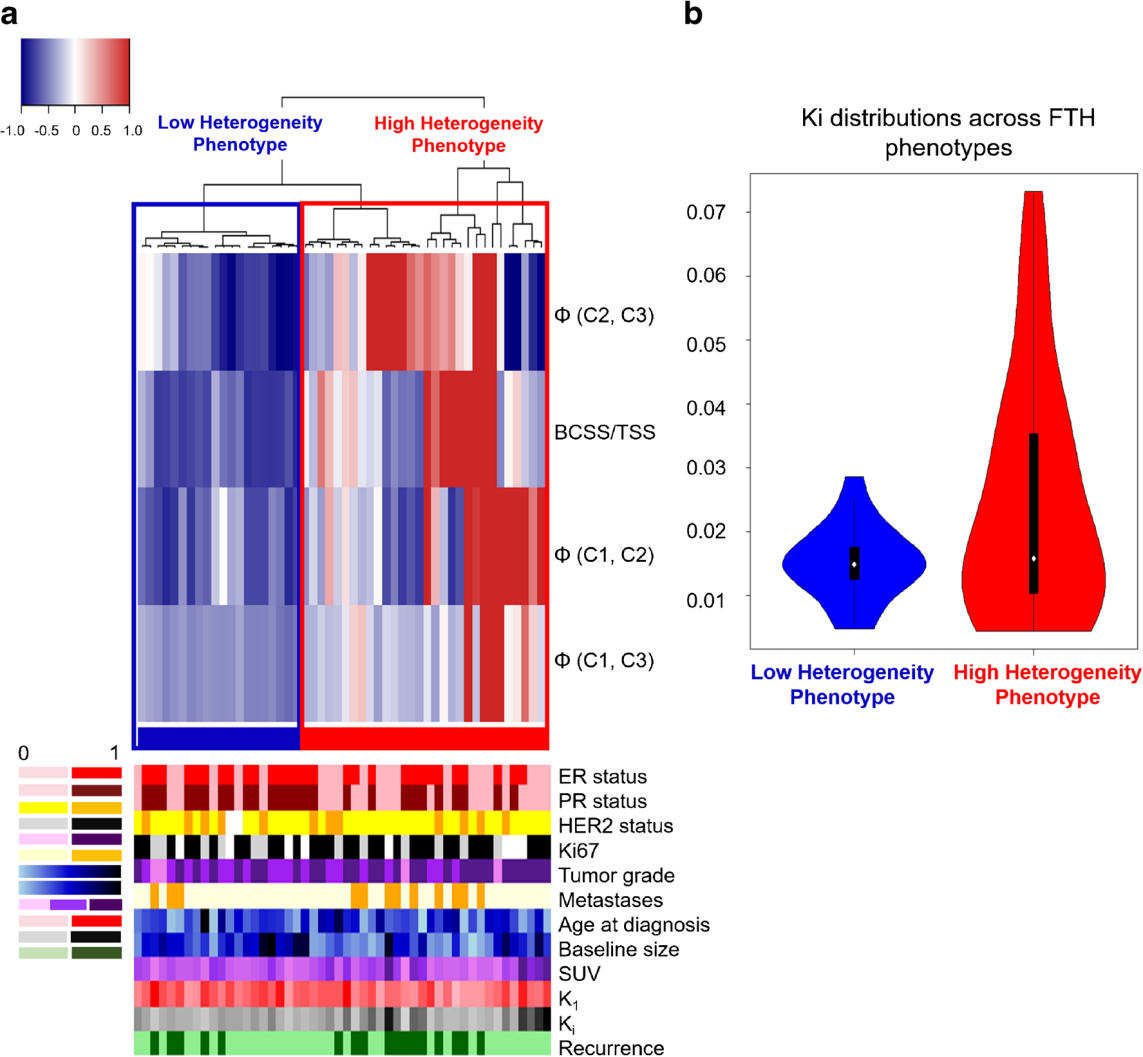
**a** Unsupervised hierarchical clustering of FTH signatures identifies 2 significant phenotypes of FTH, with clinical covariate distribution across identified phenotypes displayed in the bottom legend. The resulting cluster dendrogram can be seen above a heatmap in which each row represents a feature within the FTH signature, and each column represents a tumor. **b** Distributions of Ki across the identified phenotypes were found to be statistically significant (*p* < 0.05)

Unsupervised hierarchical clustering of women based on the extracted FTH signatures from each tumor identified two clusters which were interpreted as FTH phenotypes seen in the study population and found to be statistically significant via the SigClust method (*p* = 0.04) (Fig. 6a).

As a higher FTH signature index suggests greater separation between the three sub-regions’ FPC values and can therefore be interpreted as greater intratumor heterogeneity, the identified FTH phenotypes were ranked based on the mean FTH signature index found across all women assigned to each phenotype. The resulting FTH phenotypes 1 and 2 were interpreted as a low FTH versus high FTH phenotypes, respectively, with tumors in phenotype 1 having lower mean FTH signature indices (blue color in Fig. 6a), versus tumors in phenotype 2 which had on average higher FTH signature indices (red color in Fig. 6a).

Tumor PR status was found to be statistically significantly different across the two FTH phenotypes (*p* < 0.05), with tumors in the low FTH phenotype having a higher proportion of PR-positive tumors. Other clinical covariates including ER status, HER2 status, Ki67 status, pCR, ALN positivity, and tumor grade were not statistically significant across identified phenotypes. From the FDG PET covariates, Ki was found to be statistically significantly different across the two phenotypes (*p* < 0.05), with tumors in the high FTH phenotype having a greater interquartile range (0.025) and greater variance (0.0003) than tumors in the low FTH phenotype (interquartile range: 0.004, variance: 3.27e-5) (Fig. 6b). K_1_ and SUV values were not found to be statistically significant across the identified phenotypes.

## Discussion

Our results suggest that incorporation of both spatial and kinetic information in a 4-D dynamic activity curve clustering paradigm allows for improved segmentation of dynamic PET imaging data over established unsupervised clustering techniques utilizing kinetic information alone. Established unsupervised voxel parcellation techniques largely assume voxel independence [5], and as such, may be inadequate for identifying spatially constrained, functionally similar sub-regions under the hypothesis that subclonal populations can occupy spatially contiguous regions with common biologic properties [36]. Along these lines, partial volume effects seen in imaging modalities suggest that neighboring voxels may share information regarding underlying tissue structure due to the spatial limitations of the imaging device [37]. Therefore, analyzing imaging presentations of intratumor heterogeneity requires a fully 4-D approach.

Use of simulation images of distinct regions with known spatial locations and tracer kinetics allowed for method development and evaluation and allowed for the comparison across established techniques in this exploratory analysis. Comparing the segmentation performance of Rad-FIT clustering against established unsupervised segmentation algorithms demonstrated improved Dice scores of 0.70, 0.85, and 0.86 across low, medium, and high uptake sphere segmentation, respectively, when using simulated data with pre-defined ground truth. *K*-means clustering performed the second highest when all segmentation algorithms were evaluated, resulting in Dice scores of 0.65, 0.84, and 0.84 across low, medium, and high uptake sphere segmentation, respectively (Table 1). Additionally, when segmenting the simulated image into five regions of distinct tracer uptake, the Rad-FIT clustering algorithm outperformed *K*-means clustering as demonstrated by mean percent error in average TACs from each region as well as the corresponding Dice scores and Jaccard indices. The better segmentation performance of Rad-FIT clustering emphasizes the added benefit of incorporating both spatial and kinetic information to allow for more accurate identification of functionally distinct sub-regions. As the Rad-FIT clustering algorithm outperformed the established unsupervised clustering algorithms in the simulation analysis, we chose to use this algorithm to characterize FTH as a prognostic biomarker.

We had the goal of developing methodology broadly applicable to PET tracers with similar kinetic features. With this in mind, dynamic simulations utilized for Rad-FIT development and validation were based on FLT simulated data and used to select an approach which was then applied to a previously collected FDG patient dataset to examine the role of FTH as a prognostic biomarker. The rationale was that while simulation curves were generated using kinetic parameters specific to FLT (flux between 0.03 and 0.1 mL/min/g), these kinetics curves have parameters similar to FDG PET curves (flux between 0.02 and 0.09 mL/min/g), and they can be generalized to all tracers fit to a two-compartment model, including FDG, as long as the model and range of parameters has overlap with these tracers. Additionally, and as our algorithm is in principle agnostic to the type of tracer used or any related kinetic modeling parameters, utilizing simulated images of a different two-compartment radiotracer during Rad-FIT development allowed for a more generalizable algorithm that was not biased towards a single specific tracer in subsequent analyses.

Extending the Rad-FIT clustering algorithm to characterize intratumor heterogeneity has the potential to identify intratumor sub-regions with discrete functional behavior. This is supported by the average TACs from the identified sub-regions in representative tumors (Fig. 4), where the tumor with disease recurrence clustered into three sub-regions with distinct curve patterns. The tumor with no disease recurrence and characterized as ER+, demonstrated mostly low uptake and non-rising curves in the identified sub-regions.

Quantifying intratumor heterogeneity using the FTH imaging signature demonstrates prognostic value when predicting RFS. Cox-regression models incorporating FTH signatures added to a baseline model demonstrated a statistically significant improvement in *C*-statistic. While the dichotomization of baseline risk scores based on known prognostic features did not demonstrate significant Kaplan-Meier curve separation in this relatively small sample size, consistent with previous analyses of this study cohort [9, 22], improvement over models combining baseline and kinetic features emphasizes the added prognostic value of utilizing quantitative features summarizing dynamic tumor behavior over the entire volume. Additionally, risk scores generated using the baseline features and FTH signature resulted in statistically significant patient dichotomization into low- and high-risk groups for RFS using Kaplan-Meier survival analysis as compared to risk signatures generated from the baseline model and baseline plus kinetic model.

While prior studies have shown prognostic value for measures obtained from serial dynamic FDG PET using standard kinetic analysis methods (10), pre-therapy FDG dynamic data were not significantly predictive. Similarly, prior studies demonstrated predictive value pre-therapy measures of FDG flux and tumor blood flow obtained from combined ^15^O-water PET and FDG PET studies [21, 22], but pre-therapy FDG kinetic measures alone were not significantly predictive of RFS. In this preliminary analysis, use of the Rad-FIT clustering algorithm extracted significantly prognostic 4-D signatures from pre-therapy dynamic FDG PET data that did predict RFS, a notable incremental improvement on standard approaches to dynamic PET analysis of considerable potential significance.

Additionally, our results suggest that intrinsic imaging phenotypes may exist within locally advanced breast tumors corresponding to FTH. In particular, statistically significant differences in the FDG flux constant, *K*_i_, were seen across the two phenotypes with tumors corresponding to higher degrees of FTH having higher values of *K*_i_. This finding suggests that the tumor characteristic of increased metabolic rate may be captured within the FTH imaging signature generated from the 4-D clustering performed using Rad-FIT and may have prognostic significance when expanded to a larger study cohort. Interestingly, compared to tumor clinical and histopathologic features, we found significant differences across the FTH phenotypes in PR expression, a marker shown to be an indicator of tumor ER functionality and a more differentiated breast cancer biologic phenotype [38].

Limitations of our study should be noted. First the Rad-FIT clustering algorithm utilizes *K*-means clustering as an initialization to the method, which can allow for sensitivity to cluster initialization due to *K*-means clustering identifying local optima. Future studies will be conducted to evaluate segmentation performance when random cluster initializations are selected. Additionally, our study utilized a relatively small sample size of patients. To account for potential model overfitting, we utilized threefold CV in our time-to-event analysis, to ensure model robustness. The identification of FTH phenotypes within the study population is limited by a lack of independent validation and instead was conducted as an exploratory analysis. Future work will include expanding this initial, exploratory analysis to a larger cohort as well as validating the identified FTH phenotypes. While the Rad-FIT clustering paradigm identified three clusters within each tumor, the optimal number of functionally discrete sub-regions may vary across tumors. In this exploratory study, the selection of three for the number of subtypes was chosen empirically, guided by the three major subtypes of breast cancer (ER+/PR+, Her2+, Triple negative). Future work will also include optimization of the Rad-FIT clustering algorithm such that an optimal number of clusters is identified within each tumor. In addition, we have evaluated only the pre-therapy time point in this initial analysis. All women included in our study underwent neoadjuvant chemotherapy and repeat mid-therapy imaging. To account for the effect of treatment on intratumor heterogeneity and corresponding FTH signatures, we plan to expand our analysis to dynamic FDG PET images taken also during the midpoint of each woman’s therapy in a future study. Lastly, alternative approaches exploring a linear analysis of 4-D dynamic PET using a mixture-based approach have been previously reported [39, 40]. Future work will include expanding our analysis to compare methodologies and potentially include a mixture-based component. Lastly, we developed and applied this method on simulated dynamic images and clinical dynamic scans of breast cancer patients, with a larger goal of extending this method towards analyzing other solid tumors and different PET tracers in future work.

## Conclusion

In conclusion, we have developed a 4-D clustering and segmentation algorithm to identify functionally discrete, spatially constrained sub-regions within breast tumors that was able to generate prognostic measures from pre-therapy dynamic FDG PET of locally advanced breast cancer not previously identified by ROI-based kinetic analysis. Our results demonstrate that quantifying functional tumor heterogeneity can provide independent and additional prognostic value and may provide a non-invasive- 4-D characterization of breast tumors towards personalized decision making.

## Supplementary Information

ESM 1 (DOCX 102 kb)

## Data Availability

Custom code was generated using MATLAB and R- available upon request.
