# Correction to: Functional 4-D clustering for characterizing intratumor heterogeneity in dynamic imaging: evaluation in FDG PET as a prognostic biomarker for breast cancer

**DOI:** 10.1007/s00259-021-05324-0

**Published:** 2021-06-12

**Authors:** Rhea Chitalia, Varsha Viswanath, Austin R. Pantel, Lanell M. Peterson, Aimilia Gastounioti, Eric A. Cohen, Mark Muzi, Joel Karp, David A. Mankoff, Despina Kontos

**Affiliations:** 1 Department of Bioengineering, University of Pennsylvania, Philadelphia, PA USA; 2 Department of Radiology, University of Pennsylvania, Rm. D702 Richards Bldg. 3700 Hamilton Walk, Philadelphia, PA 19104 USA; 3 Department of Radiology, University of Washington, Seattle, WA USA

**Correction to Eur J Nucl Med Mol Imaging**

10.1007/s00259-021-05265-8

The article “Functional 4-D clustering for characterizing intratumor heterogeneity in dynamic imaging: evaluation in FDG PET as a prognostic biomarker for breast cancer”, written by Rhea Chitalia, Varsha Viswanath, Austin R. Pantel, Lanell M. Peterson, Aimilia Gastounioti, Eric A. Cohen, Mark Muzi, Joel Karp, David A. Mankoff, and Despina Kontos, was originally published electronically on the publisher’s internet portal on March 7 2021 without open access. With the author(s)’ decision to opt for Open Choice the copyright of the article changed on May 12, 2021 to © The Author(s) 2021 and the article is forthwith distributed under a Creative Commons Attribution 4.0 International License, which permits use, sharing, adaptation, distribution and reproduction in any medium or format, as long as you give appropriate credit to the original author(s) and the source, provide a link to the Creative Commons licence, and indicate if changes were made. The images or other third party material in this article are included in the article's Creative Commons licence, unless indicated otherwise in a credit line to the material. If material is not included in the article's Creative Commons licence and your intended use is not permitted by statutory regulation or exceeds the permitted use, you will need to obtain permission directly from the copyright holder. To view a copy of this licence, visit http://creativecommons.org/licenses/by/4.0/.

The original article has been corrected

