# Supplementary material for: Functional 4-D clustering for characterizing intratumor heterogeneity in dynamic imaging: evaluation in FDG PET as a prognostic biomarker for breast cancer
Source: Eur J Nucl Med Mol Imaging. 2021 Mar 7;48(12):3990–4001. doi: 10.1007/s00259-021-05265-8 (PMC8421450; doi:10.1007/s00259-021-05265-8)
Supplement: Supplementary file 1 — (DOCX 102 kb) [file 259_2021_5265_MOESM1_ESM.docx]

**Supplementary information**

**­­Title**: Functional 4-D clustering for characterizing intratumor heterogeneity in dynamic imaging: Evaluation in FDG PET as a prognostic biomarker for breast cancer

**Authors**: Rhea Chitalia^1,2^, Varsha Viswanath^1,2^, Austin R. Pantel^2^, Lanell M. Peterson^3^, Aimilia Gastounioti^2^, Eric A. Cohen^2^, Mark Muzi^3^, Joel Karp^2^, David A. Mankoff^2^, Despina Kontos^2^

^1^Department of Bioengineering, University of Pennsylvania, Philadelphia, PA.

^2^Department of Radiology, University of Pennsylvania, Philadelphia, PA.

^3^Department of Radiology, University of Washington, Seattle, WA.

**Corresponding author:**

Despina Kontos

Despina.Kontos@pennmedicine.upenn.edu

Rm. D702 Richards Bldg.

3700 Hamilton Walk, Philadelphia PA 19104

Tel: 215-746-4064

Fax: 215-573-1811

**Rad-FIT clustering**

For a given voxel ***v*** within a 3-D ROI**,** the 4-D functional behavior can be represented as *v*_(x,y,z,y,t)_ given a 3-D spatial location x,y,z and *t* representing the temporal signal from a set of dynamic frames. The temporal signal of an ROI over dynamic PET scans is first summarized using functional principal component analysis (FPCA), with each voxel represented using functional principal components (FPC) capturing greater than 85% of the variance seen in its dynamic behavior (*v*_x, y, z, f_ ). Use of FPCA allows for the dimensionality reduction of time series data for each voxel while retaining the variance of its temporal information.

A Markov Random Field (MRF) segmentation paradigm is applied to the voxel data. Here, ignoring the independence assumption between image voxels, the prior probability of a label *z* for pixel *v*_x,y,z,f_  is then modeled using a simple state prior model (Eq 1) [1].

$p\left( z_{x,y,z}=k | z^{l}, l\in N_{x,y,z} \right)= \frac{e^{\beta\delta_{x,y,z\left( k \right)}}}{\sum_{k=1}^{K} e^{\beta\delta_{x,y,z\left( k \right)}}}$ (1)

where *k* is the number of possible labels, β is an floating parameter controlling the influence of neighboring voxels, and *N_x,y,z_* defined as a 3x3x3 voxel grid surrounding *v*_x,y,z,f_. The conditional distribution of *v*_x,y,z,f_ is modeled as a multivariate Gaussian distribution (Eq 2).

$p\left( v_{x,y,z,f}) \right|z_{x,y,z}=k , \phi)=\frac{1}{2\pi^{m\backslash2} \left| \Sigma_{k} \right|^{\frac{1}{2}}}e^{-\frac{1}{2}\left( v_{x,y,z,f}-\mu_{k} \right)^{T}\Sigma_{k}^{-1}\left( v_{x,y,z,f}-\mu_{k} \right)}$ (2)

Initialization of mean and standard deviation values for each label *k* is performed using K-means clustering with a predetermined number of *k* labels. The posterior distribution for all labels, *k*, and all *n* pixels within the ROI is modeled by,

$$p\left( z_{x,y,z}^{t}=k \right|v_{x,y,z,f})= \frac{p\left( v_{x,y,z,f} | z_{x,y,z}^{t}=k \right)P\left( z_{x,y,z}^{t}=k \right)}{\Sigma^{n}p\left( v_{x,y,z,f} | z_{x,y,z}^{t}=k \right)P\left( z_{x,y,z}^{t}=k \right)}$$

 (3)

and parameters $\theta^{\left( t+1 \right)}$ are updated by the following equations (Eq 4, Eq5):

$$\mu_{k}^{t+1}= \frac{\Sigma^{n}p\left( z_{x,y,z}^{t}=k | v_{x,y,z,f} \right)v_{x,y,z,f}}{\Sigma^{n}p(z_{x,y,z}^{t}=k |x_{i)}}$$

 (4)

$\left( \sigma_{kf}^{2} \right)^{t+1}=\frac{1}{m}\frac{\Sigma^{n}p\left( z_{x,y,z}^{t}=k | v_{x,y,z,f} \right)\left( v_{x,y,z,f}-\mu_{kf}^{t+1} \right)^{2}}{\Sigma^{n}p\left( z_{x,y,z}^{t}=k | v_{x,y,z,f} \right)}, f=1,2,..m$ (5)

where *j* is the number of FPCs selected. The optimization problem is then solved using the expectation maximization algorithm [2].

**Table 1.** Radiomic functional intratumor (Rad-FIT) clustering

|  |
| --- |
| **Algorithm 1 — Radiomic functional intratumor (Rad-FIT) clustering** |
|  |
| **Input: V_i_** ∈ R*^3^*^×^*^t^*,  $\beta$ (floating parameter), *K* (number of subregions) |
| **Output:**  **z** ∈ [0,1]*^K^* (Clustering Assignment) |
| **Initialization:** Initialize **z,** $\boldsymbol{\mu,\sigma}$ by K-means clustering algorithm  **Step 1:** Functional principal component analysis (retain components with 85% of variance: **V_(x,y,z,t)_** » **V_(x,y,z,f)_** |
| **Loop-** Repeat (t) until convergence |
| • Fix **z,** $\boldsymbol{\mu,\sigma}$ — Solve for  $p\left( v_{x,y,z,f}) \right\vert z_{i}=k ,\boldsymbol{\mu,\sigma})$ |
| • Fix $p\left( v_{x,y,z,f} \vert z_{x,y,z}^{t}=k \right)$ — Solve for $z^{t}=\underset{z \in Z}{argmax} \left[ P\left( v_{x,y,z,f} \right\vert z_{x,y,z}, \boldsymbol{\mu,\sigma}^{t} \right)P(Z)]$ |

**Table 2.** Selected study cohort characteristics

|  | **Non-recurrent cases (**n=33,66%) | **Recurrent cases**  (n=17, 34%) |
| --- | --- | --- |
| **Age** |  |  |
| 30-39 | 5 (15%) | 6 (35%) |
| 40-49 | 14 (43%) | 6 (35%) |
| 50-59 | 13 (39%) | 1 (6%) |
| 60-69 | 1 (3%) | 3 (18%) |
| 70-79 | 0 (0%) | 1 (6%) |
| **Histologic subtype** |  |  |
| Infiltrating ductal | 31 (94%) | 16 (94%) |
| Infiltrating lobular | 2 (6%) | 1 (6%) |
| **Receptor status** |  |  |
| Estrogen Receptor (ER) Positive | 19 (58%) | 10 (59%) |
| Progesterone Receptor (PR) Positive | 17 (52%) | 10 (59%) |
| Human Epidermal Growth Factor Receptor 2 (HER2) | 6 (18%) | 6 (35%) |
| **Receptor subgroup** |  |  |
| ER + /HER2 + | 2 (6%) | 5 (29%) |
| ER+/ HER2 - | 16 (48%) | 4 (24%) |
| ER- /HER2 + | 2 (6%) | 3 (18%) |
| ER-/HER2- (Triple negative) | 11 (33%) | 5 (29%) |
| **pCR** |  |  |
| Complete response | 8 (24%) | 3 (17%) |
| **ALN positivity** |  |  |
| Max, min, average | 18, 0 , 2.26 | 18, 0 , 5.52 |
| **Baseline tumor size** |  |  |
| Max, min, average (cm) | 11, 1.1, 5.2 cm | 10, 1.9, 4.9 cm |
| **Ki67 status** | 27 cases | 14 cases |
| Low | 2 (7%) | 4 (27%) |
| Intermediate | 2 (7%) | 5 (33%) |
| High | 23 (85%) | 7 (47%) |

**Table 3.** Average segmentation performance over ten replicates evaluated using the Jaccard index when segmenting low, medium, and high uptake simulated sphere regions from surrounding backgrounds. Standard deviation in parentheses.

|  |  | **Jaccard indices** | | | |
| --- | --- | --- | --- | --- | --- |
| **Segmentation region** | **Voxel representation** | **Hierarchical clustering** | **Spectral clustering** | **K-means clustering** | **Rad-FIT clustering** |
| Low uptake sphere | Time activity curves | 0.08 (0) | 0.01 (0.01) | 0.07 (0.03) | 0.14(0.05) |
|  | FPC | 0.50 (0) | 0.09 (0.03) | 0.55 (0.2) | 0.54 (0) |
| Medium uptake sphere | Time activity curves | 0.64 (0) | 0.015(0.02) | 0.30 (0.37) | 0.58 (0) |
|  | FPC | 0.65 (0) | 0.12 (0.17) | 0.72 (0.04) | 0.74 (< 0.01) |
| High uptake sphere | Time activity curves | 0.56 (0) | 0.52 (0.04) | 0.44 (0.36) | 0.52 (0) |
|  | FPC | 0.71 (0) | 0.08 (0.01) | 0.73 (0) | 0.75 (0) |

**Table 4.** Risk of breast cancer recurrence in Hazzard Ratios (HR) associated with baseline model

| **Covariate** | **HR** | **95% CI** | **p-value** |
| --- | --- | --- | --- |
| ER status | 1.17 | 0.24-9.98 | 0.87 |
| PR status | 0.51 | 0.05-4.8 | 0.55 |
| Tumor size | 0.99 | 0.82-1.22 | 0.99 |
| pCR | 1.12 | 1.01-1.21 | 0.01 * |
| ALN positivity | 0.85 | 0.23- 3.15 | 0.81 |

**Table 5.** Risk of breast cancer recurrence associated with FTH imaging signature adjusting for baseline and kinetic features

| **Covariate** | **HR** | **95% CI** | **p-value** |
| --- | --- | --- | --- |
| BCSS/TSS | 1.08 | 0.62-1.89 | 0.77 |
| ϕ(1,2) | 0.04 | 0.002-0.66 | 0.02* |
| ϕ(1,3) | 0.82 | 0.48-1.39 | 0.46 |
| ϕ(2,3) | 14.08 | 2.41-21.18 | 0.003* |


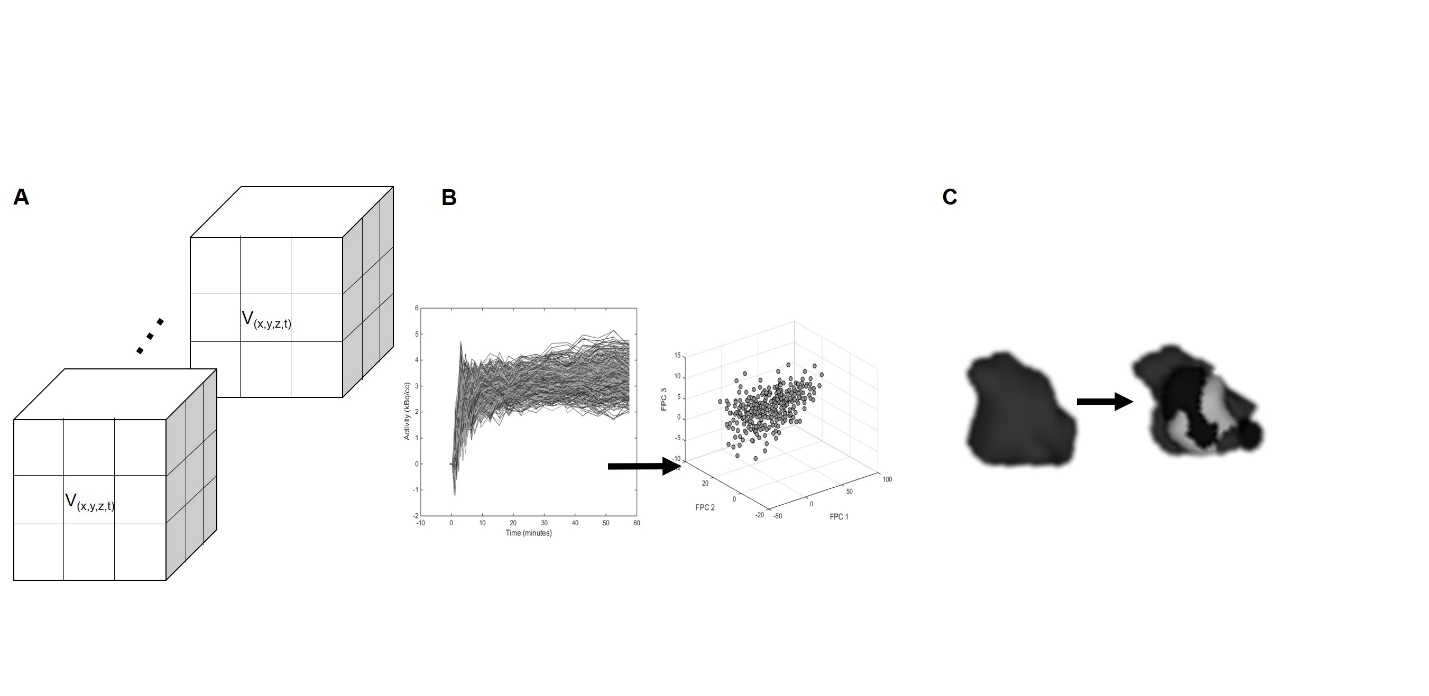


**Figure 1**: Rad-FIT clustering algorithm. (A) Give a voxel V(x,y,z,t) over a dynamic scan the time activity curve is generated. (B) Time activity curve information is summarized using functional principal component analysis, with voxels represented as functional principal components V(x,y,z,f). (C) Markov random field clustering applied to identify K subregions.

**Functional tumor heterogeneity (FTH) signature extraction**

The resulting Rad-FIT clustering within each tumor was summarized using metrics describing sub-region compactness and separation. Compactness was measured using the between cluster sum of squares (BCSS) scaled by the total sum of squares (TSS):

$\frac{\sum_{k=1}^{K} \left( \bar{x_{k}}- \bar{X} \right)^{2}}{\sum_{i=1}^{N} \left( x_{i}- \bar{X} \right)^{2}}$ (6)

where K represents the 3 sub-regions and N is the total number of voxels within each tumor. The separation between sub-regions was determined using the Bhattacharya distance [3] to calculate the distance between FPC distributions of two sub-regions, represented as ϕ and defined as:

$\phi(p,q) = -ln (\sum_{x \in X} \sqrt{p(x) q(x)})$ (7)

Use of this distance allows for a similarity measure between the distributions of FPC values within two sub-regions.

Based on the definitions above, a total of four features summarizing intratumor heterogeneity from Rad-FIT clustering results were extracted to form an FTH signature (Figure 2): (1) BCSS/TSS, (2) distance between sub-region 1 and 2 (ϕ(1,2)), (3) distance between sub-region 2 and 3 (ϕ(2,3)), and (4) distance between sub-region 1 and 3 (ϕ(1,3)). Calculation of BCSS/TSS provides a measure of how compact the resulting clusters are; the more compact each cluster is, the greater heterogeneity between the identified sub-regions. Calculating the distance between the distributions of FPC values of two sub-regions provides a metric for how separated the clusters are; a greater distance between sub-regions indicates greater heterogeneity within the whole tumor region. This FTH signature can be used to interpret how distinct the three identified sub-regions are within each tumor. As such, the average value of the FTH signature, or FTH signature index, can provide a metric for intratumor heterogeneity across tumors.

1. Chen JL, Gunn SR, Nixon MS, Gunn RN. Markov random field models for segmentation of PET images. Biennial International Conference on Information Processing in Medical Imaging: Springer; 2001. p. 468-74.

2. Dempster AP, Laird NM, Rubin DB. Maximum likelihood from incomplete data via the EM algorithm. Journal of the Royal Statistical Society: Series B (Methodological). 1977;39:1-22.

3. Bhattacharyya A. On a measure of divergence between two statistical populations defined by their probability distributions. Bull Calcutta Math Soc. 1943;35:99-109.
